# Antibiotics Use in Hospitalised COVID-19 Patients in a Tertiary Care Centre: A Descriptive Cross-sectional Study

**DOI:** 10.31729/jnma.7394

**Published:** 2022-07-31

**Authors:** Bibechan Thapa, Samyam Bickram Pathak, Nisha Jha, Milesh Jung Sijapati, Pathiyil Ravi Shankar

**Affiliations:** 1 Department of Emergency Medicine, Kirtipur Hospital, Kirtipur, Kathmandu, Nepal; 2 Department of Intensive Care Unit, Nepal Mediciti Hospital, Bhaisepati, Lalitpur, Nepal; 3 Department of Clinical Pharmacology, KIST Medical College and Teaching Hospital, Mahalaxmi, Lalitpur, Nepal; 4 Department of Internal Medicine, KIST Medical College and Teaching Hospital, Mahalaxmi, Lalitpur, Nepal; 5 IMU Center for Education, International Medical University, Bukit Jalil, Kuala Lumpur, Malaysia

**Keywords:** *antibiotics*, *bacterial infection*, *co-infection*, *COVID-19*

## Abstract

**Introduction:**

Antimicrobial resistance is a global health problem. The widespread and improper antibiotics use is the leading cause of antimicrobial resistance. Bacterial co-infection in COVID-19 patients is the basis for the use of antibiotics in the management of COVID-19. COVID-19 pandemic has seriously impacted antibiotic stewardship and increased the global usage of antibiotics, worsening the antimicrobial resistance problem. The use of antibiotics among COVID-19 patients is high but there are limited studies in the context of Nepal. This study aimed to find out the prevalence of antibiotic use among hospitalised COVID-19 patients in a tertiary care centre.

**Methods:**

A descriptive cross-sectional study was conducted on hospitalised COVID-19 patients from April 2021 to June 2021 in a tertiary care centre. Ethical approval was taken from the Institutional Review Committee (Reference number: 2078/79/05). The hospital data were collected in the proforma by reviewing the patient's medical records during the study period of 2 months. Convenience sampling was used. Point estimate and 95% Confidence Interval were calculated.

**Results:**

Among 106 hospitalised COVID-19 patients, the prevalence of antibiotics use was 104 (98.11%) (95.52-100, 95% Confidence Interval). About 74 (71.15%) of patients received multiple antibiotics. The most common classes of antibiotics used were cephalosporins, seen in 85 (81.73%) and macrolides, seen in 57 (54.81%) patients.

**Conclusions:**

The prevalence of antibiotics use among hospitalised COVID-19 patients was found to be higher when compared to other studies conducted in similar settings.

## INTRODUCTION

Antimicrobial resistance (AMR) is a major threat to global public health due to the increasing incidence of resistant human pathogens.^1,2^ The widespread and improper use of antibiotics is the leading cause of AMR.^1^ Coronavirus Disease 2019 (COVID-19) is a viral disease thus untreatable by antibiotics, but the viral respiratory infections may clinically progress to bacterial pneumonia requiring antibiotic administration.^2^ This co-pathogenesis is the basis for use of antibiotics in COVID-19. But appropriate use of antibiotics is utmost to prevent AMR.

COVID-19 pandemic has seriously impacted antibiotic stewardship and single-handedly increased the global usage of antibiotics, causing a cascading effect on the AMR problem. The use of antibiotics among COVID-19 patients is high but there are limited studies in the context of Nepal.^3-5^

This study aimed to find out the prevalence of antibiotics use among COVID-19 patients of a tertiary care centre.

## METHODS

A descriptive cross-sectional study was conducted at KIST Medical College and Teaching Hospital after taking ethical approval from the Institutional Review Committee (Reference number: 2078/79/05). The study was conducted during the study period from 6 August 2021 to 6 October 2021 during which hospitalised COVID-19 patients admitted from April 2021 to June 2021 were studied. All the COVID-19 cases confirmed by reverse transcriptase polymerase chain reaction (RT-PCR) test who were admitted in the dedicated COVID-19 ward, high dependency unit (HDU) and intensive care units (ICU) were enrolled. Patients who had incomplete documentation were excluded from the study. Convenience sampling was used. The sample size was calculated using the following formula:


$$\begin{array}{ll} \text{n} & ={\text{Z}}^{2}\times \frac{\text{p}\times \text{q}}{{\text{e}}^{\text{2}}}\\ & =1.96^{2}\times \frac{0.50\times 0.50}{0.10^{2}}\\ & =97 \end{array}$$

Where,

n = minimum required sample size
Z = 1.96 at 95% Confidence Interval (CI)
p = prevalence taken as 50% for maximum sample size calculation
q = 1-p
e = margin of error, 10%

Minimum sample size calculated was 97. However, we enrolled 106 cases. The collected data from hospital records was entered in the proforma by reviewing the patient's medical records during the study period of two months. Demographic profile of patients like age and sex, clinical profile like co-morbidity and disease severity, management profile like level of care required for patients' treatment, number and type of antibiotics used, route of antibiotic administration, duration of antibiotics used, and estimated cost of antibiotics used for the treatment were assessed. The patients who were treated with at least one antibiotic were included. All the cases were classified as a mild disease, moderate disease, or severe disease.^6^

In our study, 17 different antibiotics were used belonging to seven different antibiotic classes. They are namely cephalosporin (ceftriaxone, cefixime, cefoperazone, and cefepime), macrolides (azithromycin, clindamycin, and erythromycin), penicillin group (piperacillin, amoxicillin), quinolones (moxifloxacin, levofloxacin, ciprofloxacin), imidazoles (metronidazole), carbapenem (meropenem) and beta-lactamase inhibitors (clavulanic acid, tazobactam, sulbactam).

The data were entered and analysed using IBM SPSS Statistics 21.0. Point estimate and 95% CI were calculated.

## RESULTS

Among 106 hospitalised COVID-19 patients, the prevalence of use of antibiotics was 104 (98.11%) (95.52-100, 95% CI). The mean number of antibiotics used per patient was 1.86 ±0.64. A total of 74 (71.15%) patients were under two or more antibiotic therapy. Around 60 (57.69%) patients were treated with intravenous as well as per oral route of administration of antibiotics.

The mean number of days of admission was 6.44±4.81 days. The mean duration of antibiotics use was 6.33 ±2.72 days. About 30 (28.85%) received a 5 day course of antibiotics while 25 (24.04%) patients received a 7 days course of antibiotics. Only 23 (22.12%) received antibiotic therapy for more than 7 days. The mean estimated expenditure on antibiotics was NPR 4,645±8, 498 (USD 38.71±70.82) (Table 1).

**Table 1:** Use of antibiotics in management of COVID-19 patients (n = 104).

Characteristics	n (%)
**Number of antibiotics used**	
1	30 (28.85)
2	59 (56.73)
3	15 (14.42)
**Route of antibiotics**	
Intravenous route only	37 (35.58)
Per oral route only	7 (6.73)
Intravenous and per oral route	60 (57.69)
**Total number of days of antibiotics use**	
≤7 days	81 (77.88)
>7 days	23 (22.12)
**Estimated expenditure on antibiotics therapy NPR (US dollar)**	
≤1,200 (USD 10)	43 (41.35)
1201 to 6,000 (USD 11 to 50)	42 (40.38)
6,001 to 12, 000 (USD 51 to 100)	8 (7.69)
12,001 to 24,000 (USD 101 to 200)	7 (6.73)
>24,000 (USD 200)	4 (3.85)

The mean age of the patients was 55.84±18 years. A total of 54 (51.92%) patients were males. Around 59 (56.73%) had at least one comorbid condition with the most common conditions being hypertension seen in 39 (37.50%) and diabetes mellitus seen in 23 (22.16%) (Table 2).

**Table 2:** Demographic characteristics of hospitalised COVID-19 patients who received antibiotic therapy (n= 104).

Age group	n (%)
≤20 years	5 (4.81)
21 to 40 years	18 (17.31)
41 to 60 years	37 (35.58)
61 to 80 years	37 (35.58)
>80 years	7 (6.73)
**Sex**	
Males	54 (51.92)
Females	50 (48.08)
**Comorbidities**	
Diabetes mellitus	23 (22.16)
Hypertension	39 (37.50)
Chronic Obstructive Pulmonary	10 (9.62)
Disease (COPD)	
Hypothyroidism	12 (11.54)
Psychiatric illness	2 (1.92)
Heart failure	2 (1.92)
Autoimmune disease	2 (1.92)
Chronic kidney disease	1 (0.96)

Severe COVID-19 represented 37 (35.58%) of total patients, 16 (15.38%) of them were managed in ICU with ventilator support. Moderate COVID-19 cases also accounted for 37 (35.58%) of total patients. These patients were mostly managed in a dedicated COVID-19 ward with 2 (1.92%) cases managed in ICU and 2 (1.92%) in HDU. All 30 (28.84%) mild cases were managed in the ward (Table 3).

**Table 3:** COVID-19 severity and level of care received by the patients who received antibiotic therapy (n = 104).

Level of care	Mild n (%)	Moderate n (%)	Severe n (%)	Total n (%)
ICU with ventilator support	-	-	16 (15.38)	16 (15.38)
ICU without ventilator support	-	2 (1.92)	4 (3.85)	6 (5.77)
HDU	-	2 (1.92)	15 (14.42)	17 (16.35)
Ward	30 (28.84)	33 (31.73)	2 (1.92)	65 (62.50)

The most common class of antibiotics used was cephalosporins in 85 (81.73%) patients followed by macrolides in 57 (54.81 %). Cefixime used in all cases was a substitute for ceftriaxone in oral form in 18 (17.31%) patients who were previously prescribed ceftriaxone. Beta-lactamase inhibitors were used in 35 (33.65%) in conjunction with penicillin (amoxicillin) or cephalosporin group of drugs (cefoperazone, cefepime). The most common combination used was cephalosporin with macrolides at 38 (36.54%) (Table 4).

**Table 4:** Types of antibiotics used in management of COVID-19 patients (n = 104).

Antibiotics	n (%)
Cephalosporins prescribed parenterally	85 (81.73)
Ceftriaxone	76 (73.08)
Cefepime sulbactam	3 (2.88)
Cefoperazone sulbactam	6 (5.77)
Cephalosporins prescribed enterally	18 (17.31)
Cefixime	18 (17.31)
Macrolides	57 (54.81)
Azithromycin	55 (52.88)
Erythromycin	1 (0.96)
Clindamycin	1 (0.96)
Penicillins	26 (25.00)
Piperacillin tazobactam	14 (13.46)
Amoxicillin clavulanic acid	12 (11.54)
Quinolones	11 (10.58)
Moxifloxacin	9 (8.65)
Levofloxacin	2 (1.92)
Ciprofloxacin	1 (0.96)
Imidazoles	10 (9.62)
Metronidazole	10 (9.62)
Carbapenems	4 (3.85)
Meropenem	4 (3.85)

## DISCUSSION

The prevalence of use of antibiotics was 98.1%. About 71.15% patients were treated with two or more antibiotics. The mean number of antibiotics used per patient was 1.86. The mean duration of antibiotics use was 6.33 days. Seventeen different antibiotics were used belonging to 7 different antibiotic classes. The most common class of antibiotics used was cephalosporin at 85 (81.73%) and macrolides at 57 (54.81%).

Even before the COVID-19 pandemic, AMR was projected to become responsible for approximately 10 million deaths worldwide in the coming three decades.^7^ COVID-19 has undoubtedly affected antibiotic stewardship and has increased antibiotic consumption patterns globally, adding to the already existing global AMR problem. Because of this, the mortality due to AMR is expected to be higher in post COVID era.^2^ This pandemic has disrupted health delivery systems worldwide. This has increased the overuse of antibiotics, eventually leading to resistant organisms requiring aggressive treatment.^8^ Thus AMR is a problem of greater concern than COVID-19 which has unfortunately been overshadowed amidst the pandemic.^7,9^ Increased use of antibiotics is more challenging, especially in the low and middle-income countries (LMIC) due to the inefficiency and inadequacy of health care services.^2^

The Infectious Diseases Society of America (IDSA) states that only 8% of the COVID-19 patients acquired bacterial/fungal superinfections requiring antibiotics.^10^ However, a study showed 72% of COVID-19 patients received empirical broad-spectrum antibiotics, even when bacterial coinfection was absent.^11^ Current World Health Organization guidelines indicate that antibiotics should not be prescribed in mild or moderate COVID-19 cases unless there are pre-existing symptoms of bacterial co-infection. Furthermore, when treating severe cases with an empirical antimicrobial agent, the overall condition of the patient, local bacterial epidemiology, and clinical judgement should be integrated, to ensure judicial antimicrobial usage.^12^ In COVID-19 patients, antibiotics are used for potential anti-inflammatory, immune-modulating, and potential antiviral properties. But the antiviral mechanism of these agents is doubtful. This widespread antibiotic use is likely to worsen preexisting AMR crisis.^13^

The influenza pandemic was largely a problem of viral infection complicated by bacterial co-pathogenesis.^14^ This has been our basis for use of a wide range antibiotics empirically though COVID-19 is primarily a viral pathology and is not conventionally treated with antibiotics.

In a study, 71.00% of the hospitalised COVID-19 patients received antibiotics despite a confirmed bacterial co-infection rate of only 1%.^3^ Antibiotic was used in 95.00% COVID-19 patients when secondary bacterial infection was only found in 15.00%.^4^ A systematic review showed the mean rate of antibiotic use was 74.00 %.^5^ In our study, the prevalence of use of antibiotics was 98.10% which is very high when compared to above studies. In most cases antibiotics use often empirical. Empiric antibiotics were often used for the concern of community-acquired pneumonia (89.00%).^15^ This showed that antibiotic therapy has been used often empirically in the majority of patients even when very few were proven to have bacterial coinfection.

In our study, 17 different antibiotics belonging to seven antibiotic classes were used. Similar to our study a wide range of antibiotics use was documented in other studies.^1,5,10,13,15-18^ Many other classes of antibiotics other than above were used in other studies for the management of COVID-19 patients. They are aminoglycosides,^1^ glycopeptide antibiotic like vancomycin and teicoplanin,^10^ oxazolidinones like linezolid, tetracycline and cyclic lipopeptides like daptomycin.^17^ Most of these are newer classes of antibiotics and increased use of these should raise a red flag among concerned clinicians, pharmacists, microbiologists, public health experts, hospitals, local authorities as well as regulatory bodies.

Carbapenem, fluoroquinolones, and aminoglycoside were highly prevalent in ICU patients.^1^ Similar to this carbapenem was exclusively used for ICU patients in our study. Other commonly used antibiotics among ICU patients were fluoroquinolones, cephalosporin, piperacillin with tazobactam, and macrolides. In general, ICU addmission compromises of a very sick patient with superadded bacterial infection and in regard to COVID-19, it comprises of severe COVID-19 infection often requiring ventilatory support. In such conditions, it is common practice to use multiple higher and broad-spectrum antibiotics.

The common antibiotics in use were ceftriaxone (54.00%), vancomycin (48.00%), azithromycin (47.00%), and cefepime (45.00%).^10^ In our study ceftriaxone (73.08%) and azithromycin (52.88%) were widely used but cefepime was used in 2.88% of patients and vancomycin was not used at all. Higher antibiotics like cefepime and vancomycin should only be used when there is a valid indication, otherwise, it may result in resistant infection which will be very hard to treat.

Ceftriaxone and azithromycin are often the most common antibiotics used in the management of COVID-19 patients.^10,13,15,16^ Most of the local guidelines as well as some international guidelines advocate for use of these antibiotics based on the epidemiology of local pathogens and resistance patterns. The advantage of the use of these antibiotics is that it covers most of the opportunistic pathogens that could cause secondary infection in COVID-19. But on the other hand, wide and inappropriate use of these antibiotics can lead to with emergence resistance of these common, cheap, and very efficient antibiotics.

Macrolide, specifically azithromycin, was the most common antibiotic used in the clinical management of COVID-19.^13^ Macrolides, particularly azithromycin, were used in the treatment of more than half of the patients in our study. These drugs are often used to cover atypical organism that have the potential to cause a secondary infection.^10,11^

Fluoroquinolones were most used, (56.80%), followed by ceftriaxone (39.50%), then azithromycin (29.10%), and carbapenems were only used in two patients.^18^ Unlike to above study, in our study fluoroquinolones were used less (10.58%) and ceftriaxone and azithromycin was basically used in most patients. But similarity was observed between ours and the above study regarding the use of carbapenems, which was used in 3.85% of patients. Wide use of carbapenem, used in up to 40.10% of patients was also reported.^5^ carbapenem is often used as a reserved antibiotic for severe infection thus minimal use of these in COVID-19 signifies the presence of good antibiotics stewardship and antibiotic management system among concerned institutions while wide use can signify the opposite.

The most common antibiotic used was third-generation cephalosporin (ceftriaxone) (53.80%), moxifloxacin (29.50%), and doxycycline (25.40%).^5^ Similar to the above study the most common class of antibiotics used in our study was cephalosporin (81.74%), around 90.00% of which was third-generation cephalosporin namely ceftriaxone (76/85). But unlike the above, Moxifloxacin was used in only 8.65% and doxycycline or other tetracycline group of drugs was not used at all in our study.

A study showed all patients were receiving at least one antibiotic with 31.08% receiving a single antibiotic and 68.91% receiving multiple antibiotics.^5^ Similar to this study, 98.10% of patients in our study received at least one antibiotic, 28.35% received single antibiotic agents and 71.15% received multiple antibiotics. But the mean number of antibiotics used in the study above was 2.02 which is more when compared to 1.85 in our study. In 34.6%, three antibiotics were given simultaneously while 9.6% received only one antibiotic.^16^ Unlike the above, in our study three antibiotics were used in only 14.42% and a single antibiotic was used in 28.85%. The use of multiple antibiotics is worrisome as this might represent unchecked use of antibiotics which will contribute to the worldwide problem of AMR.

In our study, patients with comorbidity were found to have received multiple antibiotics (72.90%). Around 74.00% of patients with diabetes were on multiple antibiotics. Similar findings were documented in another study.^5^ This might be due to COVID-19 patients with comorbidities like diabetes, airway diseases, hypertension being at greater risk of developing secondary bacterial infection.^19^ After predicting the risk of secondary infection, multiple antibiotics were used empirically in those patients.

Overall, patients presenting with severe disease received more antibiotics.^5^ This is also true for our study. COVID-19 patients with co-morbidities and severe COVID-19 are the two most vulnerable groups of patients, thus multiple antibiotics have been found to be used liberally in these patients. These situations can be dealt with systematically by establishing standard antibiotic prescribing guidelines considering local pathogens and sensitivity patterns to antibiotics. This could be further reinforced by appropriate clinical knowledge, laboratory facilities, and surveillance systems.

In our study the mean duration of antibiotics treatment was 6.33 days which is nearly half when compared to 12.71 days with a range from 3 days to 23 days.^16^ Similar result was found in another study.^18^ Both of these, decreased and extended duration of antibiotic treatment might represent inappropriate and improper use of antibiotics regimen. Because both, underuse and overuse of antibiotics can result in the emergence of resistance.

Our study is a single centred study and has a small sample size. Therefore, our findings may not be generalizable to other settings.

## CONCLUSIONS

The prevalence of use of antibiotics among hospitalised COVID-19 patients was found to be higher when compared to other studies conducted in similar settings. Potential bacterial co-infection has been the basis for the use of antibiotics in the management of COVID-19 patients. The rate and number of antibiotics used for mild to moderate disease were also high. The common class of antibiotics used are cephalosporin and macrolides namely ceftriaxone and azithromycin. Higher class antibiotics were mostly used in the management of severe disease in ICU and with ventilator support. However, judicial use of antibiotics among COVID-19 patients with variable severity especially among those admitted in ICU and on ventilatory support could be promoted in order to reduce AMR during this COVID-19 pandemic. Robust antibiotic stewardship programs and surveillance systems should be implemented.
